# Incidence of Secondary Osteoarthritis after Primary Shoulder and Knee Empyema and Its Risk Factors

**DOI:** 10.3390/jpm14030264

**Published:** 2024-02-29

**Authors:** Sabrina Böhle, Luise Finsterbusch, Julia Kirschberg, Sebastian Rohe, Markus Heinecke, Georg Matziolis, Eric Röhner

**Affiliations:** 1 Orthopaedic Department of the Waldkliniken Eisenberg, Orthopaedic Professorship of the University Hospital Jena, Campus Waldkliniken Eisenberg, 07607 Eisenberg, Germany; 2 Clinic for Orthopedics, Trauma Surgery and Hand Surgery Sophien-und Hufeland-Klinikum, 99425 Weimar, Germany; 3 Orthopaedic Department, Heinrich-Braun-Klinikum, 08060 Zwickau, Germany; eric.roehner@hbk-zwickau.de

**Keywords:** empyema, joint empyema, knee empyema, postinfectious osteoarthritis, secondary osteoarthritis, septic arthritis, shoulder empyema

## Abstract

Empyema of the joint is an orthopedic emergency that is associated with a prolonged healing process despite adequate surgical and medical therapy. The risk of developing postinfectious osteoarthritis (OA) after successfully treated joint empyema is unknown. Both incidence and risk factors are important for prognostication and would therefore be clinically relevant for the selection of an adequate infectious therapy as well as for the individual follow-up of patients. The aim of this retrospective clinical study was to describe the risk of secondary OA after empyema based on knee and shoulder joint infections after successful primary infection treatment and its risk factors. Thirty-two patients were examined clinically and radiographically after completion of treatment for primary empyema of the knee or shoulder joint. Patients with previous surgery or injections in the affected joint were excluded from the study. The cumulative incidence of new-onset radiographic OA was 28.6%, representing a 5.5-fold increased risk of developing OA compared to the normal population. A figure of 25% of patients underwent total knee arthroplasty after knee empyema. Identified risk factors for primary empyema were obesity, hyperuricemia, and rheumatoid arthritis. Only about 60% of the patients tested positive for bacteria. Staphylococcus aureus, the most common pathogen causing joint empyema, was present in approximately 40% of cases. Secondary osteoarthritis, as a possible secondary disease after joint empyema, could be demonstrated and several risk factors for the primary empyema were identified.

## 1. Introduction

Septic arthritis occurs in the general population at a rate of 4 to 10 per 100,000 people per year [1]. The knee is the most commonly affected joint, followed by the hip and shoulder joints [2,3]. Most commonly, septic arthritis is caused by hematogenous dissemination of pyogenic pathogens in the setting of an underlying inflammatory disease. Another important pathomechanism is iatrogenic transmission through joint puncture, local injection, or arthroscopy [4,5]. Joint empyema is an orthopedic emergency and often leads to significant cartilage destruction within a short period of time, resulting in high medical costs for the healthcare system [6,7]. Therefore, immediate treatment of the infection should be performed, as the chances of recovery without complications depend on the stage of the disease [8]. Gächter’s classification, which is currently the most widely used, distinguishes four degrees of severity based on arthroscopic and radiographic joint damage [9]. The extent of therapeutic measures depends on the staging with antibiotic therapy [10] and surgical intervention [11,12,13].

It is known that the incidence of septic arthritis in patients with rheumatoid arthritis is up to 10 times higher than in the normal population [14]. Pre-existing diabetes mellitus also influences the development of joint infections [15]. Older patients are significantly more likely to be affected [1,4]. An increased incidence of a joint infection has also been described in immunosuppressed patients [16]. Previous surgical procedures on the affected joint and the presence of prosthetic material increase the risk of bacterial colonization and the development of empyema [15].

The identification of the pathogen and the preparation of a resistogram are crucial for the diagnosis and further therapy in order to initiate a targeted antibiotic therapy and to be able to take appropriate surgical measures. However, despite the presence of a bacterial infection, pathogen identification is not successful in all cases. *Staphylococcus aureus* is the most common cause of arthritis [17]: it can be detected in about half of all cases of septic arthritis, followed by coagulase-negative *staphylococci* [2].

The prognosis of septic arthritis is highly dependent on the stage at the time of diagnosis and the exhaustion of therapeutic options. Elimination of the infection as soon as possible is of paramount importance [18]. However, the longer the infection persists, the more likely it is that the cartilage layer will be affected [19]. Delayed initiation of therapy therefore increases morbidity, which can lead to cartilage and bone destruction as well as pathologic dislocation or stiffening of the joint [8]. Overall, an average of 40% of patients have permanent joint dysfunction [4].

The risk of developing postinfectious secondary osteoarthritis (OA) after successfully treated joint empyema is not known. The aim of this study is to investigate whether the incidence of OA after successful primary treatment of knee and shoulder joint infections differs from that in the general population, and to proof already known risk factors for empyema.

## 2. Materials and Methods

### 2.1. Patient Population

Only patients with primary joint empyema were included in this monocentric retrospective study, as it was assumed that the joint was already damaged in the case of secondary empyema. Inclusion criteria were primary joint infections of the knee or shoulder in patients who underwent arthroscopic surgical treatment of the infection between 2001 and 2013. Exclusion criteria were previous surgery, such as arthroscopy with meniscus surgery or cruciate ligament surgery, or puncture of the affected joint for secondary infection.

### 2.2. Study Implementation

The study was approved by ethics committee of the University Hospital Jena, Germany (No. 4050-04/14). All procedures were performed in accordance with relevant guidelines and regulations. The written informed consent for participation, patient information, and images to be published was provided by the patients. For all included patients, in addition to name and gender, age at surgery, affected joint, comorbidities, height, and weight were recorded to calculate body mass index (BMI). Microbiological findings were obtained from patient records. To assess radiographic changes in the joints, radiographs obtained during the clinical follow-up of the patients were evaluated according to the criteria of Kellgren and Lawrence and compared with the preoperative radiographs [20]. The preoperative radiographs of 4 patients were not available, so only the radiographs of 28 patients were evaluated. Moreover, joint replacements were noted. The following outcomes were collected during the clinical follow-up examination, or missing scores were sent by post: 36-Item short-form survey (SF36 score), Western Ontario and McMaster Universities Osteoarthritis Index (WOMAC), and visual analog scale (VAS) in all patients; Lysholm knee score and Knee Society score (KSS) in knee patients; and Constant score in shoulder patients. 

### 2.3. Statistical Evaluation

Both the collection of data and the preparation of graphs and tables were performed using the Microsoft Office Excel 2007 12.0 program (Microsoft, Redmond, WA, USA). The significance calculation for the comparison of risk factors was performed using the Chi-square test. A *p* value < 0.05 was set as the significance level. Calculations were performed using the Chi-square Calculator from Social Science Statistics (Chi-Square Test Calculator (https://www.socscistatistics.com/tests/chisquare2/default2.aspx (accessed on 13 November 2020))). The unpaired *t*-test was used to compare the results of the functional scores. Calculations based on mean, standard deviation, and population size were performed using GraphPad’s calculator (GraphPad Software, San Diego, CA, USA).

## 3. Results

### 3.1. Baseline Demographics

A total of 32 patients with primary empyema were included in the study: 25 males (78%) and 7 females (22%). The mean age at surgery was 58.8 ± 13.5 years (23–79 years). The knee joint was involved in 24 cases and the shoulder joint in 8 patients. In 22 patients, more than one operation was required to treat the joint infection. Patients were followed for 16 to 185 months after empyema (mean 88.9 months). The following comorbidities were detected: arterial hypertension (44%), diabetes mellitus (19%), hypercholesterolemia (16%), hyperuricemia (10%), rheumatoid arthritis (6%), and osteoporosis (3%). At least overweight (BMI > 25 kg/m^2^) was present in 27 cases (93%). Overall, 53.6% of participants in the study had pre-obesity and 39.3% had manifest obesity with a BMI > 30 kg/m^2^. Germs were detected in 59.38% of cases, with *Staphylococcus aureus* being the most common predominant germ in 13 of 19 patients with positive germ detection (68.42%). No bacteria were detected in 13 cases (40.63%).

### 3.2. Radiological Progression of Arthrosis according to Kellgren and Lawrence Stages

**Figure 1:**
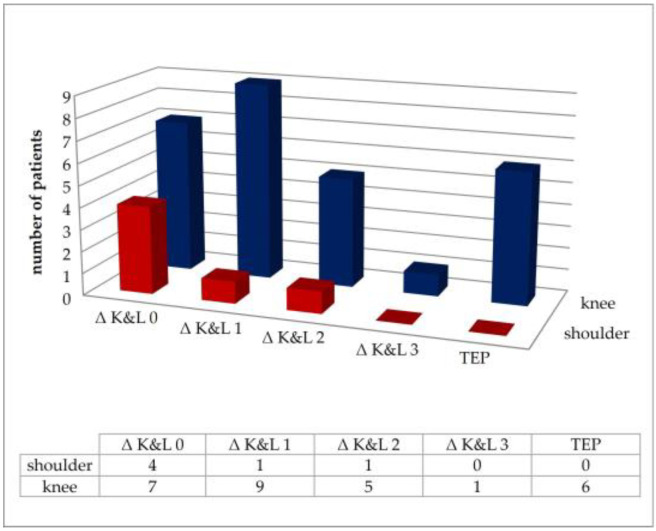
Progression of osteoarthritis stages, according to Kellgren and Lawrence, as the difference between the stage at the time of empyema and follow-up, and representation of the number of prostheses.

A total of 14 of 28 patients already had OA at the time of empyema (50%), as radiologically confirmed OA is already present from stage 2 [20]. At follow-up, a total of 22 patients had stage ≥2 (78.6%). In seven cases, stage 4 was present (25%). No radiographic progression was observed in 11 patients (39.3%). The eight shoulder patients showed less progression of radiographic OA criteria. In four of them, there was no radiologic evidence of progression (Figure 1).

### 3.3. Endoprosthetic Joint Replacement

In 6 of 32 patients, joint replacement was performed between the time of empyema and our follow-up (4 men, 2 women). This included only patients with knee joint empyema, resulting in a cumulative incidence of 25% in a total of 24 knee patients. The mean time between empyema and total knee arthroplasty (TKA) was 59.6 months (14–150 months). At clinical follow-up, patients had no extension or flexion deficit or other postoperative complications such as thrombosis or periprosthetic infection after TKA. None of the patients with shoulder empyema had a prosthesis implanted at follow-up.

### 3.4. Scores

The following Table 1 shows the evaluation of the SF-36:

The WOMAC score yielded a mean of 3.4 ± 4.4 in the “pain” subscale, 1.8 ± 2.4 in “stiffness”, 13.8 ± 15.0 in “function”, and an overall score of 19.0 ± 20.0. A total of 30% of the patients surveyed had a total score of 0. The visual analog scale yielded a joint-specific mean score of 31 ± 31 mm.

The evaluation of the Lysholm score yielded a mean of 73 ± 25 points. Very good function scores of >90 points were present in nine patients. However, a total of 10 patients (45%) had scores ≤65, indicating severe limitation in daily living [21].

The KSS knee averaged 86.1 ± 16.3 points in our study. The function score, which is the patient’s subjective perception of functional limitation in everyday situations, was 79.8 ± 19.2 points (standard deviation 19 points). The sum of KSS knee and function averaged 165.9 ± 33.5 points. The minimum was 101 points, while four patients reached the maximum of 200 points.

The Constant score used to assess the shoulder patients averaged 83.75 ± 17 points (range 53–109). 

## 4. Discussion

The aim of this study was to determine the risk of developing postinfectious secondary OA after successfully treated joint empyema, and to clarify potential risk factors for primary empyema. It is well known that there is a significantly increased risk of OA of the knee joint after trauma [22]. Wilder et al. described a nine-fold increased risk of developing secondary knee osteoarthritis after previous trauma [23]. In the US population, 12% of symptomatic OA was due to previous trauma to the affected joint [24]. Detailed studies on the relationship between joint infections and the resulting development of secondary OA are not known. In our study, 14 patients already had radiologically confirmed OA (Kellgren and Lawrence stage II) at the time of empyema. Thus, the prevalence of OA at disease onset was 50%. At the time of follow-up, 22 patients had radiographic evidence of OA. The cumulative incidence of secondary OA after empyema was thus 28.6% (8 of 28 patients). Of 24 patients with knee joint empyema, 6 underwent joint replacement during follow-up. Thus, the cumulative incidence of TKA after empyema was 25%. In the literature, the prevalence of TKA has been reported to be 1.52% in the general US population and as high as 4.55% in people over 50 years of age [25]. Compared to the general population, our study thus demonstrated a 16-fold increased risk of TKA after empyema. Taking into account the mean age of the study participants of 59 years, this results in a 5.5-fold increased risk. In their prospective study, Kaandorp et al. described a poor outcome after empyema in 33% of cases. However, this included amputation, arthrodesis, or severe functional limitations in addition to endoprosthetic joint replacement. Furthermore, this study did not differentiate between primary and secondary infections [26]. In a study by Ferrand et al., approximately 6% of patients underwent joint replacement after empyema. However, the follow-up time point here was shorter than in our study, with a mean of 17 months after empyema, and all joints were included [27]. Abram et al. investigated the outcome of primary knee empyema in a retrospective cohort study. Approximately 9% of patients had received a prosthetic joint 15 years after infection. These numbers are lower than those in our own study. However, the authors also showed that the risk of requiring total knee arthroplasty was significantly increased in patients with empyema compared to the normal population. In their study, there was a six-fold increased risk [28]. The reasons for the different incidence of OA between the knee and shoulder joints are most likely to be found in the biomechanical differences in joint loading: the knee joint, as part of the body’s load-bearing axis, is subject to high weight-bearing, in contrast to the shoulder.

The most feared complication of TKA is periprosthetic infection, which occurs in approximately 1–3% of cases [29]. However, no clear clinical, microbiological, or treatment-specific risk factors for reinfection in patients with joint replacement after previous empyema have been identified to date [30]. No specific postoperative complications, in particular thrombosis/embolism or periprosthetic infection, occurred in the six TKA patients we studied. A study by Bettencourt et al. showed that the risk of PJI was increased 6.1-fold in patients with TKA and a history of native septic knee arthritis compared to patients without TKA, with a cumulative incidence of 9% after 10 years, especially in the first 5 to 7 years after diagnosis of septic arthritis [31]. In comparison, hip prosthesis patients with a history of septic arthritis are known to have a ten-fold increased risk of infection compared to normal osteoarthritis patients, with a cumulative ten-year incidence of 7% [32]. At the time of follow-up, the mean range of motion was 120° of flexion. None of the patients had an extension deficit. In studies of postoperative flexion after TKA in general, the range of flexion was usually reported to be between 100° and 115° [33]. In comparison, our patients had a very good range of motion. Bauer et al. also examined patients with TKA after previous joint infection, and also found very good functional results without exact ROM data [30]. Even in patients who had already developed complete or partial ankylosis of the knee joint as a result of the infection, it was possible to achieve ranges of motion of over 100° ROM after TKA implantation [34].

### 4.1. Pathogen Spectrum and Germ Detection

In our 32 patients, no pathogen was detected in approximately 40% of cases. This is in agreement with other studies in which no pathogen could be isolated in 38% of cases [18]. Reasons for the lack of pathogen detection include antibiotic therapy already started before the samples were preserved for microbiological examination [2]. In some cases, the pathogenic germs are not detectable in the joint puncture, but are detectable, for example, in the blood culture [35] or in tissue samples taken intraoperatively [36]. In our study, we did not distinguish whether the germs were detected in the preoperative joint punctate, from blood cultures, or from intraoperative specimens. *Staphylococcus aureus* was detected in 68.42% of the patients with a positive germ detection. Ryan et al. detected 40.6% *staphylococci* [37]. There have been no significant changes in the overall bacterial spectrum in patients with joint empyema over the past decades [38]. 

### 4.2. Scores

#### 4.2.1. SF-36

For comparison of the SF-36, the results of the DEGS1 study of the Robert Koch Institute were used (n = 8152) [39].

The comparison in Table 2 shows that patients with joint empyema have significantly worse scores than the general population, especially in the subscales representing physical health. In the mental health domains, similar or even better results are shown, such as in the subscale of psychological well-being. Thus, after a joint infection, physical functional limitations in daily life seem to persist. Mental health does not seem to be affected by the disease in the long term.

#### 4.2.2. WOMAC (Knee Joint)

No data are available on WOMAC scores in the average healthy population, particularly in age-matched individuals, with a focus on knee and shoulder joints. To compare the results in Table 3, a study by Kahn et al. was used that evaluated the clinical and radiographic outcomes of 172 patients before and after TKA. The mean follow-up was 3 years [40].

All subcategories were between the pre- and postoperative values of total knee arthroplasty patients. It can be concluded that the functional outcome after empyema is indeed better than in patients with advanced gonarthrosis. However, limitations in daily life due to pain, stiffness, and limited functional capacity of the affected joint are greater than in patients after successful total joint arthroplasty.

#### 4.2.3. VAS (Knee and Shoulder Joint)

According to Jensen et al., the pain was in the “mild” range with 31 ± 31 mm [41]. About one-third of the respondents reported no more pain. With regard to the results of the VAS in the German normal population, data are only available on general health status and not on the pain level of individual body parts [42], so that a direct comparison is not possible here. Nevertheless, it can be said that persistent pain can remain as a residual after joint empyema, which requires prolonged or permanent therapy and can also have an impact on the patient’s quality of life, as can be seen from the results of the SF-36 subscale “Pain”.

#### 4.2.4. Lysholm (Knee Joint)

The mean Lysholm score in our study was 73 ± 25 points. Demirdjian et al. examined the results of the Lysholm score in subjects without pre-existing knee disease. The results were 99 ± 3 in men and 97 ± 5 in women [43]. Patients with knee joint empyema had significantly worse joint function than healthy patients (*p* < 0.0001).

#### 4.2.5. KSS (Knee Joint)

For the comparison in Table 4, a study by Brandes et al. examined the functional outcome of patients before and in defined time periods after a total knee arthroplasty [44].

Compared to the patients before TKA, i.e., with advanced knee osteoarthritis, the empyema patients show significantly better scores (*p*-value of all three scores < 0.0001). It appears that the results of the empyema patients are most similar to the results 6 months after TKA. However, the results 12 months after TKA are significantly better than those after empyema for both total and knee scores (*p*-values < 0.0001 and 0.0036, respectively). No significant difference was found for the functional score (*p* = 0.474).

#### 4.2.6. Constant Score (Shoulder Joint)

Table 5 shows the evaluation of the Constant score in patients with shoulder joint empyema compared to healthy subjects. Data are available from Yian et al. on the results of the Constant score in healthy subjects, subdivided by sex and age group [45].

The three women with shoulder empyema scored an average of 78 ± 28 points, while healthy women in the same age group scored 85 ± 4 points. The shoulder function of the women was thus significantly worse after empyema than in the healthy normal population (*p* = 0.0344). The five men with shoulder empyema had an average score of 87 ± 10 points. The difference to the healthy population in the age group with 90 ± 6 points was not statistically significant (*p* = 0.2876). But these results should be handled with care because of the small number of patients.

### 4.3. Risk Factors for Primary Joint Empyema

Relevant comorbidities were recorded in the patients with joint empyema and compared with their prevalence in the normal German population (Table 6). When available, the prevalence of the diseases in the comparable age group was used for comparison (mean age of our study: 58.8 years).

#### 4.3.1. Obesity

In our study, 53.6% of the study participants had pre-obesity and 39.3% had manifest obesity with a BMI > 30 kg/m^2^ (a total of 92.9% with a BMI > 25 kg/m^2^). In comparison, 54.0% of the German normal population has a BMI > 25 kg/m^2^ [46]. Thus, the incidence in our patient population was significantly higher than in the normal population, confirming obesity both as a risk factor (*p*-value = 0.000037) and, at the same time, the most common comorbidity among the study participants. Grotel et al. described a significantly increased risk of knee osteoarthritis with a BMI > 30 kg/m^2^ [53], and Hochberg et al. also demonstrated a high correlation between increased body weight and knee osteoarthritis [54]. In our study, there was no relevant difference between knee osteoarthritis and shoulder osteoarthritis with respect to BMI.

#### 4.3.2. Diabetes Mellitus

Approximately 19% of patients reported underlying diabetes mellitus type I or II. In the total German population, the prevalence is 9.8% [47]. Thus, the prevalence in our patient population was almost twice as high, but not statistically significant (*p*-value = 0.088592). Thus, diabetes mellitus could not be confirmed as a risk factor in our study. Ferrand et. al. found a similarly high prevalence of 29.3% in patients with empyema of the large joints, in their study [27]. Hunter et. al. showed that the presence of diabetes mellitus increases the risk of a complicative course of empyema with the need for multiple surgical procedures [55]. OA is also commonly associated with diabetes mellitus. Veronese et al. demonstrated a pathogenetic link between the diseases. Thus, chronic hyperglycemia and insulin resistance lead to both oxidative stress and chronic low-grade inflammation in the joints, which further promote cartilage degradation and thus the progression of OA. In the aforementioned study, the prevalence of OA in diabetic patients was found to be 14% [56].

#### 4.3.3. Arterial Hypertension

Of the patients studied, 14 had pre-existing arterial hypertension (44%). According to the GEDA study of the RKI, the prevalence of arterial hypertension ever diagnosed by a physician in the German population as a whole is 31.8% [48]. There was no significant difference between the two groups (*p* = 0.146622). Arterial hypertension has not been reported as a risk factor for empyema in the literature, but studies show an association between cardiovascular disease, including arterial hypertension, and OA [57,58].

#### 4.3.4. Hypercholesterolemia

In our patient population, 15.6% of patients reported pre-existing hypercholesterolemia. According to the DEGS study, 65.12% of the German normal population had dyslipidemia [49]. With a *p*-value of <0.00001, the difference is clearly significant and can possibly be interpreted as a protective factor for empyema. However, in the DEGS, almost 60% of the subjects were unaware of their dyslipidemia at the time of the study. Therefore, a limitation of the study is that the survey of comorbidities was based solely on patient history and not on laboratory chemistry.

#### 4.3.5. Hyperuricemia

In the survey, approximately 9% reported underlying hyperuricemia. Since the prevalence in the general population is 1.4% [50], a *p*-value of 0.000123 indicates a significantly higher incidence among the 32 empyema patients. Thus, hyperuricemia was confirmed as a risk factor for joint empyema. In their cohort study, Lim et al. described a 2.6-fold increased risk of joint empyema in patients with pre-existing gout [59]. Several studies also describe the co-occurrence of crystal arthropathy with septic arthritis [60].

#### 4.3.6. Rheumatoid Arthritis

Rheumatoid arthritis is considered a risk factor for the development of joint empyema, as both chronic synovial inflammation and altered joint structure favor bacterial colonization [15]. Compared to the normal population, with a prevalence of 1.08% [51], patients with empyema had a significantly higher prevalence of rheumatoid arthritis, at 6.25% (*p*-value: 0.004662). Thus, this comorbidity was confirmed as a risk factor. Other studies on septic arthritis show an even higher prevalence of rheumatoid arthritis, with 13–14% [38].

#### 4.3.7. Osteoporosis

Among the patients with empyema we examined, only one subject reported pre-existing osteoporosis. This corresponds to about 3% of the collective and is significantly lower than the German average, with a prevalence of 11.9% according to the GEDA study [52]. The difference is not significant, with a p-value of 0.135808. There is also the limitation that this was based only on anamnestic data and not on the basis of an objective examination.

#### 4.3.8. Gender

Males were significantly more commonly affected than females in our study (*p*-value: 0.001463). Thus, male gender is a risk factor for primary joint empyema. While some studies confirm this observation [2,37], Gupta et al. found a predominance of female subjects [61]. The differences in these studies were not statistically significant. The situation is different in OA. Here, female sex is considered a risk factor. Women are 1.5 to 4 times more likely to have OA than men [62].

#### 4.3.9. Osteoarthritis

In our study, although OA was considered as a possible consequence after joint empyema had occurred, it is also one of the risk factors for the development of empyema. In this study, 50% of patients had manifest OA at the time of empyema, which was higher than in comparative studies. In the study by Cooper and Cawley, OA was present in the affected joint at the time of empyema in 20% of patients and was therefore considered a risk factor [63]. In an American study, approximately 12% of empyema patients were diagnosed with pre-existing OA [64]. However, there was no evidence that these patients had more rapid progression of OA after empyema.

In general, pre-existing chronic inflammatory joint disease predisposes to infection, although the underlying pathomechanisms are not yet fully understood. It is possible that the abnormal joint structure favors pathogenic microorganisms not being completely eliminated by phagocytes [65]. In animal studies, *staphylococci* were injected into healthy and pre-damaged joints, and significantly more severe and rapid histologic changes were demonstrated in the pre-damaged joints. As a possible reason for the more rapid joint destruction, it has been suggested that infection by pannus can spread more rapidly to the subchondral bone in arthritic joints [66]. In our study, there was no statistically significant evidence as to which patients had more or less progression of OA, as measured by Kellgren and Lawrence stages. One reason for this may be the small number of patients studied.

### 4.4. Limitations

The retrospective, monocentric design of the study has some limitations, such as incomplete documentation. Data collection by means of self-administered questionnaires always carries the risk of misreporting. In addition, this information is based on medical history alone and was not objectively confirmed or further investigated, e.g., by laboratory chemistry. The scores were only collected at the time of follow-up and could therefore only be interpreted to a limited extent. However, the reliability is limited due to the small number of patients examined, particularly those with shoulder joint empyema. This is partly due to the rather strict inclusion criteria of primary empyema without prior surgery or puncture. In addition, some patients were no longer available.

## 5. Conclusions

This retrospective clinical study demonstrated a significantly increased incidence of OA in patients with previous knee empyema compared to the normal population. Patients showed negligible to modest OA progression following shoulder empyema. Knee patients also had an increased risk of requiring total joint replacement. Obesity, male gender, hyperuricemia, and rheumatoid arthritis were confirmed as risk factors for the development of primary joint empyema. The increased risk of developing secondary OA after knee empyema should be addressed in patient education and should be monitored regularly. Although periprosthetic infection did not occur after total knee arthroplasty in the patients studied in our study, the literature suggests that patients with a history of empyema are at increased risk and should be closely monitored postoperatively for clinical and paraclinical signs of infection.

## Figures and Tables

**Table 1:** Overview of SF-36 results by subscales.

	Mean	Standard Deviation	Minimum	Maximum
Physical Functioning	63.4	29.2	0	100
Role-physical	62.5	43.0	0	100
Bodily Pain	60.8	31.1	0	100
General Health	55.9	19.9	15	87
Vitality	58.3	19.8	15	100
Social Functioning	83.8	24.4	12.5	100
Role-Emotional	75.9	42.8	0	100
Emotional Well-Being	73.9	16.3	28	100
Physical Health Scale	39.3	12.4	15.4	58.0
Mental Health Scale	52.5	9.2	26.4	59.5

**Table 2:** Comparison of results SF-36 with the respective mean values with 95% confidence interval [39].

	General Population	Empyema Study
Physical Functioning	85.2 (84.0–86.4)	63.4 (52.7–74.0)
Role-physical	80.1 (78.7–81.6)	62.5 (46.6–78.4)
Bodily Pain	72.0 (70.4–73.6)	60.8 (49.7–72.0)
General Health	67.6 (66.5–68.7)	55.9 (48.6–63.2)
Vitality	61.8 (60.6–62.9)	58.3 (51.1–65.5)
Social Functioning	84.7 (83.4–86.0)	83.8 (75.0–92.5)
Role-Emotional	84.7 (83.3–86.1)	75.9 (60.3–91.4)
Emotional Well-Being	72.0 (70.9–73.1)	73.9 (68.0–79.9)
Physical Health Scale	50.4 (49.9–51.0)	39.3 (34.5–44.1)
Mental Health Scale	49.1 (48.4–49.8)	52.5 (49.0–56.1)

**Table 3:** Presentation of the mean values with standard deviation of the individual WOMAC categories of our patients after knee empyema, and of the patients before and after implantation of a total knee arthroplasty [40].

WOMAC	Empyema	Preoperative before TKA	Postoperative before TKA
Pain	3.4 ± 4.4	6.4 ± 3.7	1.9 ± 2.6
Stiffness	1.8 ± 2.4	3.1 ± 1.8	1.4 ± 1.4
Function	13.8 ± 15.0	20.4 ± 12.8	7.5 ± 9.6
Global	19.0 ± 20.0	29.8 ± 16.9	10.8 ± 13.2

**Table 4:** Comparison of KSS of empyema patients to patients before and after TKR, mean (±SD) [44].

KSS	Empyema	Before TKA	2 Months after TKA	6 Months after TKA	12 Months after TKA
Total	165.9 ± 33.5	88.9 ± 21.4	139.3 ± 19.0	168.1 ± 17.4	188.6 ± 10.9
Knee	86.1 ± 16.3	31.6 ± 14.9	72.3 ± 11.8	84.2 ± 10.2	94.2 ± 6.0
Function	79.8 ± 19.2	57.2 ± 10.8	67.0 ± 10.9	83.9 ± 10.9	94.5 ± 8.3

**Table 5:** Comparison of the results of the Constant score of patients with shoulder joint empyema in comparison to healthy subjects [45].

	Men		Women	
	Empyema	General Population	Empyema	General Population
Number	5	127	3	101
Age	63	61–70	49	41–50
Constant score	87 ± 10	90 ± 6	78 ± 28	85 ± 4
*p*-value	0.2876		0.0344	

**Table 6:** Comparison of the occurring risk factors in patients with joint empyema with the prevalence in the normal population.

	Patients with Empyema	General Population	*p*-Value
Obesity/pre-adiposity	92.85%	54.00% [46]	0.000037
Diabetes	18.75%	9.80% [47]	0.088592
Arterial hypertension	43.75%	31.80% [48]	0.146622
Hypercholesterolemia	15.62%	65.12% [49]	<0.00001
Hyperuricemia	9.38%	1.40% [50]	0.000123
Rheumatoid arthritis	6.25%	1.08% [51]	0.004662
Osteoporosis	3.13%	11.90% [52]	0.135808

## Data Availability

The datasets used and/or analyzed during the current study are available from the corresponding author on reasonable request.
